# Translational drugs targeting cancer stem cells in triple-negative breast cancer

**DOI:** 10.1016/j.omton.2025.201008

**Published:** 2025-06-13

**Authors:** Felipe P. de Oliveira, Mateus L. Nogueira, Alexandre F.C. Galvão, Rosane B. Dias, Daniel P. Bezerra

**Affiliations:** 1 Gonçalo Moniz Institute, Oswaldo Cruz Foundation (IGM-FIOCRUZ/BA), Salvador, Bahia 40296-710, Brazil; 2 Department of Biological Sciences, State University of Feira de Santana, Feira de Santana, Bahia 44036-900, Brazil

**Keywords:** triple-negative breast cancer, cancer stem cells, cell signaling pathways

## Abstract

Triple-negative breast cancer (TNBC) is defined by the lack of expression of estrogen receptor (ER), progesterone receptor (PR), and human epidermal growth factor receptor 2 (HER2). It is unresponsive to targeted therapy and is associated with a high degree of malignancy, a high propensity for metastasis, high recurrence rates, and poor prognosis. In the modern concept of cancer biology, a subset of cancer cells known as tumor-initiating cells or cancer stem cells (CSCs) are defined as essential for the development and dissemination of cancer. These are a population of highly tumorigenic and self-renewing pluripotent cells that are inherently associated to the initiation, dissemination, relapse, and development of drug resistance. Specifically, some cell signaling pathways may affect the ability of CSCs to self-renew, differentiate, proliferate, and survive. To guide future research, in this review, we address compounds that target cell signaling and eliminate TNBC stem cells. Potential translational inhibitors of the Hedgehog, nuclear factor κB (NF-κB), Wnt, Notch, Hippo, TGF-β, JAK/STAT, and PI3K/AKT/mTOR cell signaling pathways are discussed, with a focus on TNBC stem cell eradication.

## Graphical abstract

**Figure:**
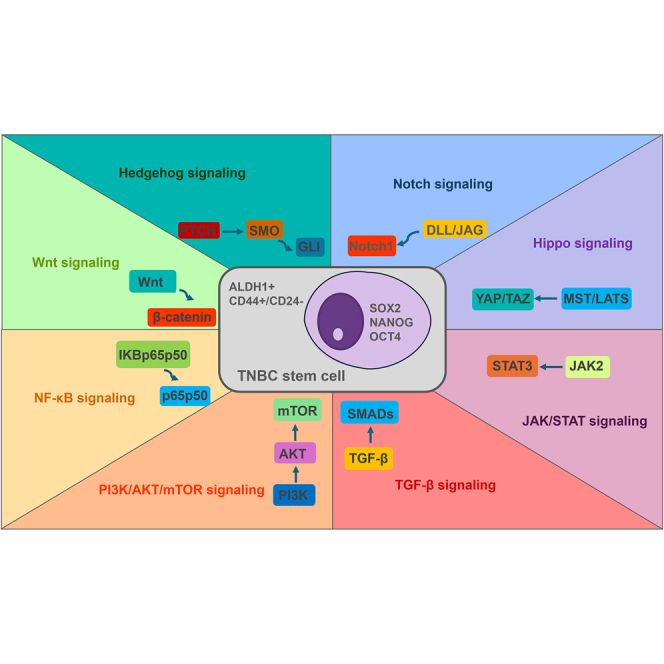

## Teaser

Triple-negative breast cancer (TNBC) has one of the highest rates of metastasis and recurrence, which has been associated with cancer stem cells (CSCs). This review explores potential translational inhibitors of Hedgehog, NF-κB, Wnt, Notch, Hippo, TGF-β, JAK/STAT, and PI3K/AKT/mTOR signaling pathways, with a focus on TNBC eradication by inhibiting CSCs.

## Introduction

Triple-negative breast cancer (TNBC) is characterized by the absence of estrogen receptor (ER), progesterone receptor (PR), and human epidermal growth factor receptor 2 (HER2) expression. This subtype of breast cancer is associated with a high degree of malignancy, propensity for metastasis, high recurrence rates, and poor prognosis.^1^^,^^2^^,^^3^ Unlike other breast cancer subtypes, TNBC does not respond to standard hormonal or HER2-targeted therapy, making it more challenging to treat.^3^ In this context, TNBC patients are treated with classical cytotoxic chemotherapies, which are associated with severe side effects and are not effective in many cases. Recently, the use of the PARP inhibitors talazoparib and olaparib has increased the number of TNBC treatment options.^4^^,^^5^

TNBC accounts for approximately 10%–15% of all breast cancers. TNBC occurs more frequently in black women under the age of 40 years and with a *BRCA1* mutation.^6^ In the United States of America, women diagnosed with TNBC between 2012 and 2018 had 5-year relative survival rates of 91% for localized stage tumors, 66% for regional stage tumors, and only 12% for distant stage tumors.^6^

According to the modern concept of cancer biology, the decisive role in cancer formation and growth is played by a subpopulation of cancer cells called cancer stem cells (CSCs) or tumor-initiating cells (TICs). These are a population of highly tumorigenic and self-renewing pluripotent cells that are intrinsically linked to the initiation, dissemination, relapse, and development of drug resistance.^7^^,^^8^^,^^9^^,^^10^^,^^11^

Some surface markers have been shown to be able to identify and isolate TNBC stem cells, including CD44,^12^ CD24,^12^ epithelial adhesion molecule (EpCAM, also known as ESA),^12^ aldehyde dehydrogenase 1 (ALDH1),^13^ CD133,^14^ and ATP-binding cassette subfamily G member 2 (ABCG2)^15^ (Table S1). On the other hand, this cellular subpopulation displays significant heterogeneity, with the reported presence of at least two distinct phenotypic and functional states: epithelial-like and mesenchymal-like states. Epithelioid breast CSCs are characterized by the prevalence of ALDH^+^ cells, which are located primarily in the central region of the tumor and exhibit relatively active proliferation. Conversely, mesenchymal breast CSCs consist mainly of CD44^+^CD24^−^ cells, are distributed at the tumor periphery, are in a state of static proliferation, and demonstrate strong invasive capabilities.^16^

Breast CSCs also highly express transcription factors, including SOX2,^17^ NANOG,^18^ and OCT4,^18^ which are required to maintain stemness and avoid differentiation. LGR5 expression was associated with the higher malignant potential.^19^ The high expression of ABC family transporters^20^ and the ALDH1 cytosolic enzyme^21^ confer resistance to traditional cancer therapies. Cell signaling pathways, such as the Notch, Wnt, Hedgehog (HH), nuclear factor κB (NF-κB), PI3K/AKT/mTOR, transforming growth factor β (TGF-β), JAK/STAT, and Hippo pathways, influence CSC features such as stemness, self-renewal, differentiation, proliferation, and survival.^8^^,^^9^^,^^22^

Owing to the absence of viable therapeutic targets, TNBC is treated primarily via traditional chemotherapy. Although chemotherapeutic drugs can destroy proliferating tumor cells, they are unable to effectively target CSCs, leading to the proliferation of these cells and the subsequent development of drug resistance in TNBC. Consequently, drug resistance facilitated by CSCs presents a significant challenge in the treatment of TNBC. In recent years, several cell signaling pathway inhibitors, including those that act on CSCs, have emerged as anti-TNBC agents. In this review, we discuss molecules that target cell signaling with the ability to eradicate TNBC stem cells to direct future research.

## Targeting cell signaling pathways in TNBC stem cells

### Hedgehog cell signaling pathway

HH is a highly conserved cell signaling pathway that is essential for embryonic development and plays a key role in the formation and maturation of breast tissue.^23^^,^^24^ Its deregulation is directly associated with deficiencies in fetal formation and neoplastic transformation, which lead to the development of several malignant tumors, including mammary cancer.^25^^,^^26^^,^^27^ Many studies indicate that the HH pathway is possibly related to the proliferation and differentiation of cells with pluripotency characteristics, guaranteeing their accumulation in normal and tumor tissues and thereby promoting the maintenance of stem cell populations.^27^^,^^28^

Canonically, the signaling cascade in this pathway is initiated in the primary cilium region of the cell membrane and triggers its function at the nuclear level, which depends on classic components, which include HH ligand proteins (Desert Hedgehog [DHH], Indian Hedgehog [IHH], or Sonic Hedgehog [SHH]), a cell surface transmembrane receptor Patched (PTCH), a transmembrane coreceptor-like protein Smoothened (SMO), and glioma oncogene-associated transcription factors (GLI; in the form of GLI1, GLI2, or GLI3). Among the three ligands, SHH is the most widely expressed.^27^^,^^29^

**Figure 1:**
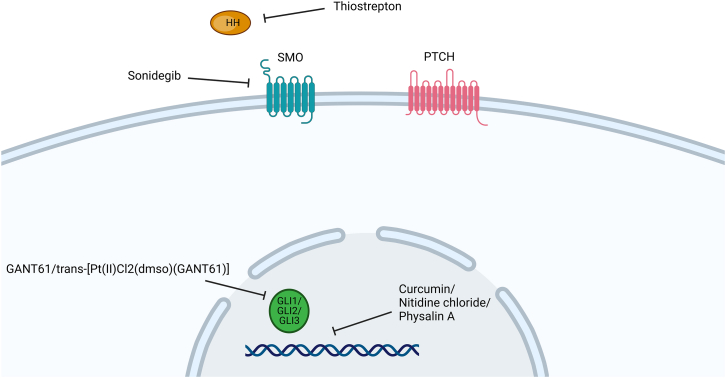
Hedgehog cell signaling pathway In the absence of Hedgehog ligand (HH), the Patched receptor (PTCH) inhibits Smoothened protein (SMO), which in turn prevents the activation of GLI transcription factors. When SHH binds to PTCH, this blockade is released, SMO is activated, and GLI factors continue to function. These proteins then translocate to the nucleus, where they activate genes involved in proliferation, differentiation, and development.

Briefly, in the presence of a binding protein released in the extracellular environment in an autocrine or paracrine manner, the PTCH receptor is inactivated, promoting the activation of SMO and its accumulation in the primary cilium. The coreceptor acts as a positive regulator of the pathway, allowing the downstream activation of a protein complex, the subsequent release of the GLI factor into the cytoplasm, its conversion into the active form and, finally, its translocation to the nucleus (Figure 1).^27^^,^^29^ GLI activates target genes, especially those responsible for controlling the cell cycle, proliferation, self-renewal, and death; in addition, it can also promote angiogenesis and regulate the epithelial-mesenchymal transition (EMT).^27^^,^^30^ In the absence of binding proteins, the PTCH receptor constitutively promotes the inhibition of the SMO coreceptor and the repression of the pathway activation signal.^29^

In turn, all alterations mediated by the activation and use of the upstream components of the HH pathway, which do not depend on the transcriptional signal usually triggered by GLI, are called noncanonical pathways.^30^^,^^31^ In this case, interactions between components of the HH pathway and other cellular signaling pathways, such as the RAS/RAF/MEK/ERK, PI3K/AKT/mTOR, and TGF-β pathways, are found.^29^^,^^32^^,^^33^

In this sense, the importance of noncanonical pathways in HH was recently reported in TNBC cells and CSC populations. The activation of GLI1 and GLI2 in TNBC cell lines occurred through a noncanonical pathway associated with the influence of NF-κB.^34^^,^^35^^,^^36^ Similarly, the use of a noncanonical inhibitor of the HH pathway resulted in a decrease in the proportion of CSCs in TNBC cell lines.^37^ These findings, associated with clinical evidence of the overexpression of HH pathway cellular components, such as SMO and GLI1, in TNBCs, which are not always correlated with a proportional increase in HH-initiating ligands, suggest that noncanonical activation is the major trigger in TNBC cells.^29^^,^^38^

In addition, some studies suggest that the HH pathway promotes an increase in the invasiveness profile and stimulation of angiogenesis in TNBC cells.^39^^,^^40^ The creation of a supportive microenvironment for cells with a pluripotency profile from the reprogramming of tumor-associated fibroblasts has also been reported.^41^^,^^42^ Furthermore, the activation of the HH pathway in tumor stem cells (CD44^high^/CD24^low^) present in populations of TNBC cells promoted their survival and clonal expansion, even after chemotherapy treatment with docetaxel.^43^ These reports highlight the crucial role played by the HH signaling pathway in the promotion of TNBC cells, especially the CSCs that compose it. They also present the opportunity to explore it as a potential target for antitumor therapies. The compounds described below have been reported to be capable of killing TNBC stem cells via HH inhibition and are discussed in this section.

#### Curcumin

Curcumin, a diphenylheptane derived from the rhizome of *Curcuma longa* L., is a low-toxicity bioagent widely used in traditional Asian cuisine and medicine.^44^^,^^45^ Its broad spectrum of pharmacological activities includes anti-inflammatory, antioxidant, antiseptic, analgesic, antiparasitic, and antitumor effects.^45^^,^^46^

Li et al.^47^ demonstrated the ability of this polyphenol to reduce the CD44^+^/CD24^-/low^ subpopulation in MDA-MB-231 cells, a TNBC cell line, after mammosphere formation in ultralow adhesion plates and culture in serum-free medium. This reduction in the number of breast CSCs was directly related to the ability of curcumin treatment to decrease the expression of genes and proteins involved in the HH pathway, such as the downregulation of SMO, GLI1 and GLI2. In addition, key genes involved in the maintenance of stem cell populations, such as those involved in stemness processes (OCT4 and SOX2) and EMT (E-cadherin and vimentin), were affected by the treatment. The inhibitory effect of curcumin was attributed to its ability to decrease the entry and accumulation of GLI1 in the nucleus of MDA-MB-231 cells. Thus, curcumin cytotoxicity operates through the HH/GLI pathway in the CSCs of TNBC cells.^47^ Furthermore, Li et al.^48^ reported that curcumin suppresses TNBC CSCs by suppressing the Wnt/β-catenin pathway in addition to the HH pathway.

A randomized, double-blind clinical trial of 150 women with advanced and metastatic breast cancer (including seven patients with TNBC) evaluated the efficacy and safety of intravenous infusion of curcumin (300 mg solution, once weekly) in combination with paclitaxel (80 mg/m^2^) for 12 weeks, with a follow-up period of 3 months. Importantly, after 12 weeks of treatment, curcumin plus paclitaxel outperformed the paclitaxel-placebo combination in terms of the objective response rate (51% vs. 33%) and physical performance.^49^ Furthermore, curcumin administration reduced doxorubicin-induced cardiotoxicity^50^ and radiation-induced dermatitis^51^ in clinical trials with breast cancer patients. Although few clinical studies have been conducted with curcumin in patients with TNBC, several other preclinical studies support the ability of curcumin to eliminate TNBC stem cells.^52^^,^^53^^,^^54^^,^^55^^,^^56^

#### Thiostrepton

Thiostrepton is a natural antibiotic isolated from bacteria of the genus *Streptomyces* and approved by the Food and Drug Administration (FDA) for veterinary use in dermatologic diseases, such as mastitis caused by gram-negative bacteria, that blocks protein synthesis by inhibiting ribosome activity in these bacteria.^57^ Its pharmacological activity has recently been tested against tumor cells, and it has been demonstrated to be promising and selective for growth inhibition, induction of apoptotic death, cell-cycle arrest, and reduction of xenographic tumors in a range of cell types.^57^^,^^58^^,^^59^ Although depletion of the Forkhead box protein M1 (FoxM1) transcription factor is reported to be the main cause of the cytotoxicity of thiostrepton in cancer, other causes have been discussed.^57^

Yang et al.^59^ demonstrated that the use of thiostrepton was able to reduce the viability of TNBC cells (MDA-MB-231 and SUM149) and decrease the proportion of the CD44^+^/CD24^−^ subpopulation in these cell lines. Similarly, the same treatment almost completely eradicated the sphere-forming ability of the TNBC cell lines studied, decreasing both the number and size of the mammospheres. The authors attributed this effect to the ability of thiostrepton to inhibit the HH pathway, specifically through the sonic ligand. In fact, their results demonstrated that treatment for 24 h significantly reduced SHH protein levels compared with those in control cells. This reduction is accompanied by a decrease in the levels of the stem cell regulator NANOG and a decrease in the expression of transcripts of downstream targets of the HH pathway, such as FoxM1, N-MYC, and CCND1. Finally, the findings were validated by silencing the gene responsible for the SHH ligand, which demonstrated that, in its absence, sphere formation is highly impacted, and the percentage of cells with a CD24^+^ phenotype is increased. These findings explain the involvement of the HH pathway in maintaining the stemness profile of TNBC cells and how the use of thiostrepton can alter this signaling pathway.^59^

#### GANT61

GANT61 is a synthetic compound derived from hexahydropyrimidine and is one of the first small molecules identified as an inhibitor of the HH pathway at the level of the GLI transcription factor.^60^ In the cell nucleus, GANT61 connects directly to GLI1 but is independent of the DNA-binding region of this transcription factor.^61^ Since their discovery, small molecules have been investigated as cytotoxic agents in several types of cancer.^60^

In the TNBC cell lines MDA-MB-231, MDA-MB-157 and HCC1937, treatment with GANT61 for 72 h decreased the proportion of stem cell subpopulations.^37^ In addition, the size of the mammospheres was also reduced after exposure to this molecule in the MDA-MB-231 and MDA-MB-157 cells. When used in combination with paclitaxel, a clinically approved drug used to treat breast cancer, GANT61 significantly reduced the number of mammospheres in all TNBC cell lines tested.^37^

#### *Trans*-[Pt(II)Cl_2_(dmso) (GANT61)]

A platinum complex with GANT61 with the formula *trans*-[Pt(II)Cl_2_(dmso) (GANT61)] was evaluated in TNBC HMLER cells, which have a CSC-like population of approximately 5%–8%, and HMLER-shEcad cells, which have a CSC-like population of approximately 90%. This complex inhibited the growth of both cell lines. Furthermore, it reduced the formation and viability of HMLER-shEcad mammospheres. DNA damage and inhibition of the HH pathway at the level of GLI were also observed.^62^

#### Sonidegib

Sonidegib, also known as erismodegib or NVP-LDE225, is a clinically approved selective SMO antagonist. Cazet et al.^42^ demonstrated that the expression of the CSC marker ALDH1 was reduced after treatment with sonidegib (80 mg/kg/day) in a long-term TNBC HCI-002 PDX model.

A phase 1 study of 18 patients with advanced solid tumors (including two patients with breast malignancies) revealed that 800 mg of oral sonidegib in combination with 80 mg/m^2^ paclitaxel is the recommended dose for a phase 2 clinical trial. The best response was partial in three patients (two with ovarian cancer and one with breast cancer), with stable disease for more than four cycles in three patients (two with ovarian cancer and one with anal cancer).^63^ In addition, a phase 1b study of sonidegib in combination with docetaxel in patients with advanced TNBC was conducted in 12 patients to explore the combination of sonidegib and docetaxel. The recommended dose for phase 2 was 800 mg of sonidegib orally once daily plus 75 mg/m^2^ docetaxel administered intravenously on the first day of a 21-day cycle. In particular, the combination indicated anticancer efficacy in 3 out of 10 patients with detectable disease.^64^

#### Nitidine chloride

Nitidine chloride is an alkaloid derived from the root of *Zanthoxylum nitidum* that has cytotoxic properties. Nitidine chloride suppressed the proliferation of the mammospheres of TNBC cells while reducing their migration and invasion through the inhibition of EMT. Nitidine chloride suppressed the expression of HH components (SMO, PTCH, GLI1, and GLI2) as well as CSC-related markers such as NANOG, Nestin, OCT4, and CD44.^65^

#### Physalin A

Physalin A is found in *Physalis alkekengi*. Physalin A caused apoptosis and growth suppression in mammospheres generated from TNBC cells, as well as a reduction in the transcript levels of CSC marker genes. The protein expression levels of SMO, GLI1/2, and YAP1 are reduced by physalin A, indicating that physalin A modulates HH and Hippo cell signaling.^66^

These data support the idea that the Hh pathway is essential for the preservation of TNBC stem cell characteristics, such as self-renewal and treatment resistance. The listed compounds target multiple aspects of this pathway, including ligand inhibition and interference with GLI transcription factors. Although the results are encouraging, further clinical trials are needed to evaluate the efficacy and safety of these compounds in humans.

### NF-κB signaling pathway

**Figure 2:**
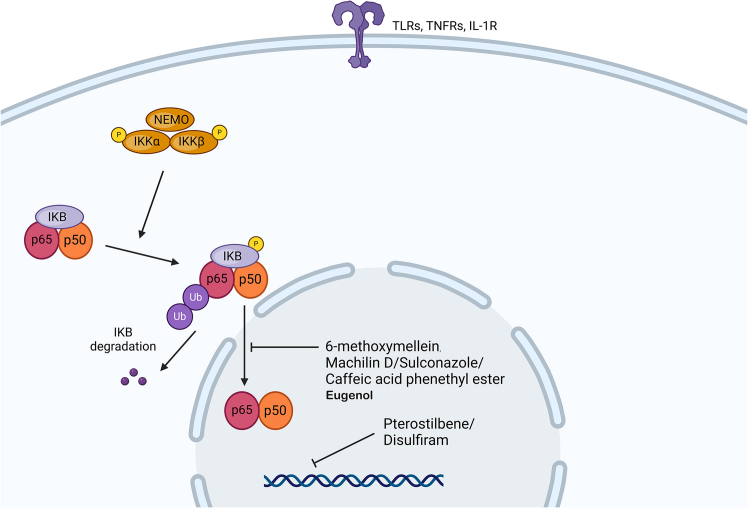
NF-κB cell signaling pathway Under resting conditions, NF-κB p65/p50 is kept inactive in the cytoplasm by the inhibitory protein IκB. Under stimuli such as cytokines or stress, IκB is phosphorylated and degraded, releasing NF-κB p65/p50, which translocates to the nucleus and increases the expression of genes related to inflammation, the immune response, and cell survival.

The family of transcription factors known as NF-κB play critical roles as stress regulators within the cellular environment. They control the expression of vital regulatory genes involved in immunity, inflammation, cell death, and proliferation. The NF-κB protein primarily resides in the cytoplasm and can be triggered by diverse cellular stimuli. The activation of NF-κB involves two pathways: the canonical pathway and the noncanonical pathway.^67^ The IKK complex, consisting of the IKKα, β, and γ subunits, activates canonical NF-κB. When IKKα is phosphorylated, it is degraded, allowing the RelA (p65) and p50 NF-κB subunits to concentrate in the nucleus and control gene transcription (Figure 2). Noncanonical signaling stabilizes NF-κB-inducing kinase (NIK), activating IKKα homodimers and cleaving the NF-κB p100 subunit. This results in an active RelB-p52 NF-κB dimer that controls transcription and translocates to the nucleus. Canonical and noncanonical NF-κB subunits can control unique or identical target genes.^68^

In cancer, NF-κB participates in the inhibition of apoptosis, promotes angiogenesis, facilitates metastasis, increases cell survival, and promotes proliferative activity. Numerous studies have demonstrated a clear correlation between NF-κB activation and the development of tumor resistance to different therapeutic agents, including radiotherapy.^69^ In TNBC cells, high levels of constitutively active NF-κB signaling are found,^70^ and mechanistically, an NF-κB-Jag1/Notch-NF-κB signaling axis promotes TNBC stem cell growth and survival.^71^ Conversely, IKKε, an NF-κB inhibitor, has been associated with increased TNBC stem cell accumulation.^72^ This section discusses drugs that have been found to suppress TNBC stem cells via NF-κB signaling inhibition.

#### Eugenol

The combination of eugenol, a naturally occurring polyphenolic compound, with cisplatin had greater cytotoxic and proapoptotic effects on TNBC cell lines both *in vitro* and *in vivo*. The combined treatment was able to decrease the growth of ALDH^+^ TNBC cells by inhibiting the NF-κB signaling pathway.^73^

#### Caffeic acid phenethyl ester

Caffeic acid phenethyl ester is an active component of propolis that inhibits NF-κB signaling. It inhibited mammosphere development in a CSC-enriched TNBC model as well as in patient-derived TNBC cells. Furthermore, in mice implanted with TNBC stem cells, this drug inhibited tumor development.^74^

#### 6-Methoxymellein

6-Methoxymellein is a chemical constituent of carrots. 6-Methoxymellein decreased the proliferation and migration of TNBC cells and suppressed the expression of the breast CSC markers c-MYC, SOX2, and OCT4, as well as the ratio of CD44^+^/CD24^−^ cells. 6-Methoxymellein inhibited the nuclear translocation of the NF-κB subunits p65 and p50 and the secretion and expression of interleukin-6 (IL-6) and IL-8.^75^

#### Disulfiram

Disulfiram is a clinically approved antialcoholism medication that inhibits ALDH activity. Disulfiram decreased the clonogenicity of breast cancer cells in copper-containing medium. Disulfiram/copper reduced mammosphere development and the population of ALDH1^+^ and CD24^low^/CD44^high^ CSCs. Increased reactive oxygen species (ROS) production, activation of the apoptosis-related JNK and p38 MAPK pathways, and decreased constitutive NF-κB activity were detected in disulfiram/copper-treated TNBC cells.^76^ Disulfiram also abolished paclitaxel resistance in TNBC cells by targeting CSCs.^77^

#### Machilin D

Machilin D is a lignin derived from *S. chinensis*. This molecule reduced the fraction of CD44^+^/CD24^−^ and ALDH1^+^ cells in TNBC, impaired growth and mammosphere formation, reduced the nuclear localization of the NF-κB protein, and decreased IL-6 and IL-8 secretion. Machilin D also reduced tumor growth in a human breast xenograft mouse model.^78^

#### Pterostilbene

Pterostilbene is a natural stilbene isolated from blueberries. Pterostilbene treatment reversed the CSC enrichment and metastatic potential of TNBC caused by tumor-associated macrophages. Pterostilbene reduced the percentage of CD44^+^/CD24^−^ MDA-MB-321 cells cocultured with tumor-associated macrophages, as well as the migratory and invasive properties of MDA-MB-231 cells. These effects are caused by the downregulation of NF-κB and EMT-associated molecules, as demonstrated *in vitro* and *in vivo*.^79^

#### Sulconazole

Sulconazole is an antifungal drug of the imidazole class. Sulconazole decreased the number of TNBC cells expressing CSC markers, such as CD44^high^/CD24^low^, ALDH, and other self-renewal genes (NANOG, c-MYC, and CD44). Furthermore, sulconazole reduced NF-κB signaling and extracellular IL-8 generation in mammospheres.^80^

These findings reinforce the importance of the NF-κB pathway for maintaining the characteristics of TNBC stem cells, such as resistance to treatment and metastasis. Despite these promising results, clinical trials are needed to determine the efficacy and safety of these drugs in humans.

### Wnt signaling pathway

**Figure 3:**
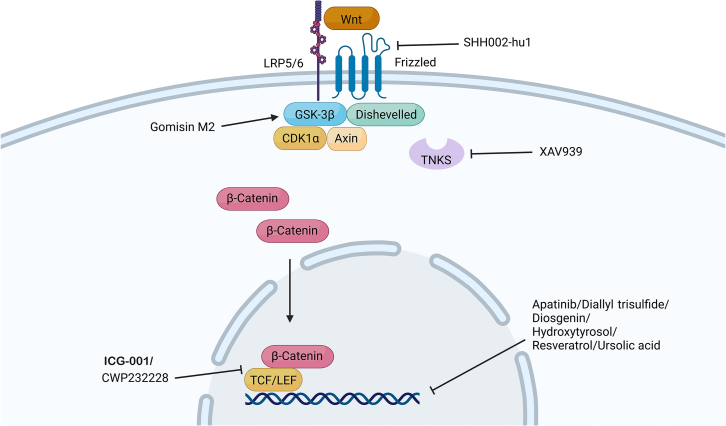
Wnt cell signaling pathway In the absence of a Wnt ligand, β-catenin is degraded by a destruction complex. When Wnt binds to the Frizzled receptor and the coreceptor LRP, this complex is inhibited, allowing β-catenin to accumulate in the cytoplasm and be translocated to the nucleus, where it activates the transcription of genes related to cell development, protection, and differentiation.

Wnt proteins are a group of proteins that are secreted and play several roles in normal cell biology and developmental processes, such as cell polarity generation and cell fate specification. Wnt signaling is initiated when secreted Wnt proteins bind to Frizzled (FZD) coreceptors and low-density lipoprotein-receptor-related proteins 5 and 6 (LRP5/6), triggering intracellular signaling cascades via β-catenin or a noncanonical pathway without β-catenin (Figure 3). In cancers, abnormal Wnt/β-catenin signaling is frequently observed, and clinical studies suggest that elevated Wnt/β-catenin signaling is linked to higher tumor grade and worse prognosis.^81^

Wnt/β-catenin signaling is associated with stem cell renewal and the differentiation of healthy epithelial cells. In breast CSCs, this signaling pathway is linked to self-renewal, mammosphere formation, cell migration, invasion, and resistance to apoptosis and radiation therapy.^81^ In fact, studies have shown that knocking down β-catenin can reduce stem-cell-like cell populations, tumor size, and doxorubicin resistance in TNBC cells.^82^

In TNBC 4T1 cells, after activation of Wnt signaling by Wnt3A treatment, the number of ALDH^+^ cells increased significantly. In contrast, inhibition of Wnt signaling reduced this subset of the cell population. Interestingly, TNBC CSCs exhibit higher levels of Wnt signaling than the main tumor population does.^83^ In this section, we explore drugs that reduce TNBC stem cells by inhibiting Wnt signaling.

#### Gomisin M2

Gomisin M2, a naturally occurring compound found in *Schisandra viridis*, is commonly used as an anticancer drug. Gomisin M2 significantly impeded the growth of TNBC cell lines and prevented the formation of mammospheres in breast CSCs by suppressing the Wnt/β-catenin self-renewal pathway. Additionally, gomisin M2 triggered apoptosis and disrupted the mitochondrial membrane potential of breast CSCs. In zebrafish, gomisin M2 restricted the growth of MDA-MB-231 and HCC1806 xenografts.^84^

#### XAV-939

XAV-939 is an indirect inhibitor of the Wnt signaling pathway. XAV-939 is a highly effective inhibitor of tankyrase (TNKS) 1 and 2 that increases the protein levels of the axin-GSK-3β complex and promotes β-catenin degradation. Christensen et al.^74^ reported that XAV-939 inhibited growth and mammosphere formation in a CSC-enriched TNBC model as well as in patient-derived primary TNBC cells.

#### ICG-001

ICG-001 is an inhibitor of β-catenin/TCF-mediated transcription, selectively blocking the β-catenin/CBP interaction. Combining ICG-001 with simvastatin, a YAP inhibitor, suppressed the CD44^high^/CD24^−/low^ mesenchymal CSC and ALDH^+^ epithelial CSC subpopulations in cultured TNBC cells and animal models. This finding suggests the requirement for dual inhibition of Wnt and YAP to suppress CSC subpopulations.^85^

#### SHH002-hu1

SHH002-hu1 is a humanized monoclonal antibody that targets Fzd7. By decreasing the TNBC stem-like cell subpopulation, SHH002-hu1 improved the ability of bevacizumab, a monoclonal antibody that blocks vascular endothelial growth factor (VEGF), to inhibit TNBC growth. This further attenuated the tumor-initiating and self-renewal capacity of TNBC cells, which was enhanced by bevacizumab. SHH002-hu1 inhibited the adaptation of TNBC cells to hypoxia by interfering with Wnt/β-catenin signaling.^86^

#### Apatinib

Apatinib, also known as rivoceranib, is a tyrosine kinase inhibitor that targets and selectively inhibits vascular endothelial growth factor receptor-2. Apatinib significantly reduced the survival of TNBC-associated stem cells and decreased colony and sphere formation, as well as inhibited migration and invasion processes. Furthermore, apatinib was found to suppress stemness characteristics, EMT, and the Wnt/β-catenin signaling pathway in TNBC stem cells. Notably, overexpression of the lncRNA ROR was able to partially counteract these effects.^87^

Importantly, apatinib has also been evaluated in clinical trials in patients with TNBC. In a phase II, open-label, noncomparative, two-arm clinical trial, 40 Chinese patients with advanced TNBC and fewer than three lines of systemic therapy were enrolled to evaluate the efficacy and safety of camrelizumab (an anti-PD-1 immune checkpoint inhibitor) in combination with apatinib. Camrelizumab (intravenously administered every other week) was combined with oral apatinib as continuous dosing (d1-d14) for 30 patients or intermittent dosing (d1-d7) for 10 patients until disease progression or intolerable toxicity. The median progression-free survival was 3.7 months in the continuous-dosing cohort and 1.9 months in the intermittent-dosing cohort, demonstrating good therapeutic benefits. The most common adverse effects are increased aspartate aminotransferase/alanine aminotransferase levels and hand-foot syndrome.^88^

Ou et al.^89^ reported that Chinese patients with stage III TNBC were enrolled in a prospective single-center phase 2 clinical study and received neoadjuvant treatment consisting of 250 mg apatinib daily, 175 mg/m^2^ paclitaxel on day 1, and carboplatin at a dose based on the area under the curve of 4 on day 2 every 14 days as one cycle. A total of 16 patients completed 4–7 cycles of apatinib treatment and 4–8 cycles of chemotherapy, resulting in 2 complete responses, 12 partial responses, and 2 stable diseases, indicating that apatinib combined with dose-dense paclitaxel and carboplatin neoadjuvant therapy is effective and well tolerated in patients with locally advanced TNBC.^89^

Zhang et al.^90^ conducted a clinical trial with 29 Chinese patients with recurrent or metastatic TNBC who received camrelizumab (200 mg every 2 weeks), apatinib (500 mg once daily), and fuzuloparib (a PARP inhibitor) (starting dose of 100 mg twice daily) every 28 days. The disease control rate was 62.1%, and the median progression-free survival period was 5.2 months, with a 12-month overall survival rate of 64.2%, indicating that these combinations have an administrative safety profile and preliminary anticancer activity.^90^

A phase 1 clinical trial of fuzuloparib in combination with apatinib was conducted in 22 patients with advanced TNBC in China. Fuzuloparib (100 mg) plus apatinib (500 mg) was defined as the highest dose with acceptable toxicity. Patients with gBRCAmut had a higher objective response rate (66.7% [2/3] vs. 15.8% [3/19]) and a longer median progression-free period (5.6 vs. 2.8 months) than those with gBRCAwt.^91^

In a single-arm phase 2 trial, 40 Chinese patients with advanced TNBC who had failed at least one course of chemotherapy were enrolled. A 3-week regimen of 500 mg oral apatinib on days 1–21 and 50 mg oral etoposide on days 1–14 was followed until disease progression or severe toxicity. The median progression-free survival was 6.0 months, and the median overall survival was 24.5 months, indicating that this combination is promising.^92^

In a multicenter phase 2 clinical trial, 46 patients with pretreated advanced TNBC received camrelizumab 200 mg (day 1) and apatinib 250 mg daily, as well as eribulin (microtubule inhibitor) 1.4 mg/m^2^ (days 1 and 8), for a period of 21 days until progression or unacceptable toxicity. This study revealed a disease control rate of 87.0% (40/46) and a median progression-free survival of 8.1 months, indicating that camrelizumab plus apatinib and eribulin has potential efficacy.^93^

A prospective, open-label, single-center, randomized, phase 2 clinical trial compared the efficacy of apatinib plus vinorelbine (33 patients) with that of vinorelbine alone (32 patients) for metastatic TNBC patients whose first- or second-line treatment failed. The median progression-free survival in the apatinib plus vinorelbine group was longer than that in the vinorelbine-alone group (3.9 months vs. 2.0 months), whereas the median overall survival was 11.5 months in the apatinib plus vinorelbine group and 9.9 months in the vinorelbine group, suggesting that apatinib combined with vinorelbine is promising for patients with advanced TNBC.^94^

Overall, preclinical research and clinical trials suggest that apatinib, particularly when combined with other drugs, may provide therapeutic benefits in patients with TNBC, including those with advanced or metastatic disease. However, phase 3 clinical trials are still needed to definitively determine the role of apatinib in the treatment of TNBC.

#### CWP232228

CWP232228 is an antagonist of β-catenin that binds to T cell factor (TCF) in the nucleus. CWP232228 inhibited the *in vitro* and *in vivo* growth of the TNBC cell line 4T1, which was especially active against TNBC stem cells through the inhibition of β-catenin-mediated transcriptional activity. Furthermore, CWP232228 was found to reduce TNBC stem cell functions, which are mediated by insulin-like growth factor I (IGF-I).^95^

#### Diallyl trisulfide

Diallyl trisulfide is a naturally occurring organosulfur compound found in garlic. Research has shown that diallyl trisulfide decreases TNBC stem cell survival, as evidenced by a reduction in tumorsphere formation and a decrease in the expression of key TNBC stem cell markers, including CD44, ALDH1A1, NANOG, and OCT4. The mechanism by which diallyl trisulfide affects TNBC stem cells involves downregulation of the Wnt/β-catenin signaling pathway. Furthermore, activation of Wnt/β-catenin with LiCl reduces diallyl trisulfide inhibition.^96^

#### Diosgenin

Diosgenin is a natural steroidal saponin that has been demonstrated to inhibit TNBC stem cell proliferation. This effect is achieved by promoting apoptosis through caspase 3/7 activation and the release of ROS, targeting the Wnt/β-catenin signaling pathway. This mechanism involves the suppression of TCF-LEF-regulated genes and a reduction in β-catenin expression. Furthermore, diosgenin decreased specific features of TNBC stem cells, including mammosphere formation and CD44 and ALDH expressions.^97^

#### Hydroxytyrosol

Hydroxytyrosol is a plant-derived molecule from olive oil that has cytotoxic potential. Tumor cell motility and invasion, ALDH^+^ and CD44^+^/CD24^−/low^ subsets, and mammary CSC self-renewal were decreased by hydroxytyrosol treatment. The downregulation of the EMT markers Slug, ZEB1, Snail, and vimentin, as well as the Wnt signaling markers LRP6, β-catenin, and cyclin D1, and the TGF-β signaling pathway marker (SMAD2/3) was detected in hydroxytyrosol-treated TNBC cells.^98^

#### Resveratrol

Resveratrol is a polyphenolic compound abundantly produced in some plant foods, such as grapes. Fu et al.^99^ reported that resveratrol reduced the proportion and prevented the growth of mammary CSCs isolated from MCF-7 and SUM159 and reduced the size and number of mammospheres. Injection of resveratrol (100 mg/kg) into NOD/SCID mice decreased the proportion of CSCs in tumor cells and suppressed the formation of xenograft tumors. Resveratrol also inhibited the Wnt/β-catenin signaling pathway and promoted autophagy.

#### Ursolic acid

Ursolic acid is a pentacyclic triterpenoid derived from several medicinal plants. Ursolic acid reduced mammary CSCs from TNBC cells through suppression of the Wnt/β-catenin pathway via upregulation of the antagonist sFRP4 and downregulation of the expression of the oncogenic miR-499a-5p.^100^

These findings indicate that the Wnt/β-catenin pathway is essential for the maintenance of stem cell characteristics in TNBC. Although the results are encouraging, further clinical trials are needed to determine the efficacy and safety of these compounds in humans. Among them, apatinib has the most advanced studies and seems to be able to be part of the treatment of TNBC in the future.

### Notch signaling pathway

**Figure 4:**
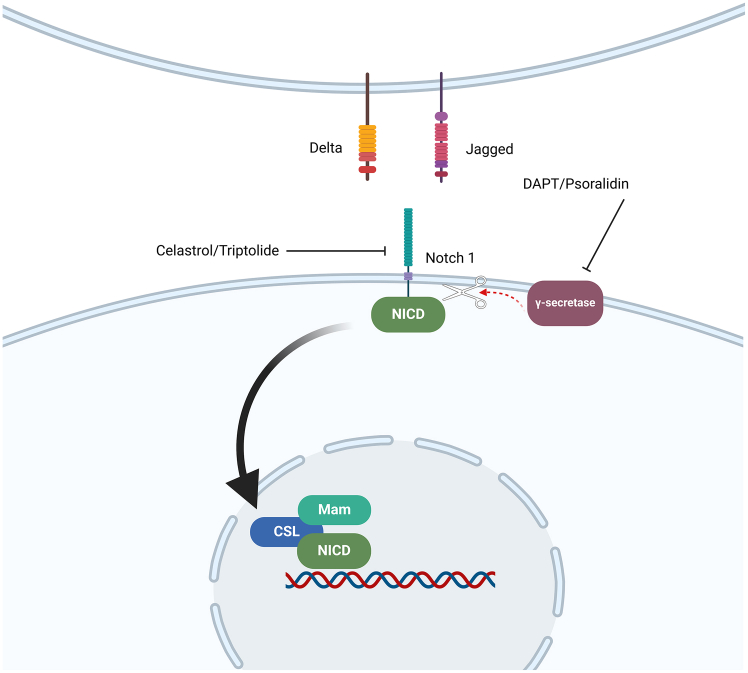
Notch cell signaling pathway Activation occurs when Notch proteins on the cell membrane interact with Delta or Jagged family ligands on neighboring cells. This leads to proteolytic cleavage of Notch and release of the intracellular domain (NICD), which translocates to the nucleus and regulates the expression of genes involved in cell differentiation, development, and tissue maintenance.

The Notch signaling pathway is critical throughout development and has been implicated in malignant transformation. When transmembrane Notch receptors bind to their ligands, such as the classical Notch ligands Delta (Dll) 1/3/4 and Jagged (JAG) 1/2, which are released by nearby cells or by themselves, the Notch signaling cascade is triggered. The Notch receptor and its ligands are transmembrane proteins with extensive extracellular domains that are predominantly composed of epidermal growth factor (EGF)-like repeats.^101^^,^^102^ Ligand interaction induces two proteolytic cleavage events at the Notch receptor. ADAM family metalloproteases catalyze the first cleavage, whereas γ-secretase, an enzyme complex that includes presenilin, nicastrin, PEN2, and APH1, mediates the second. The second cleavage releases the Notch intracellular domain (NICD), which subsequently translocates to the nucleus and collaborates with the DNA-binding protein CSL (named after CBF1, Su(H), and LAG-1) and its coactivator Mastermind (MAM) to stimulate transcription (Figure 4).^101^^,^^102^

TNBC stem cells have increased Notch signaling, which appears to be regulated by the transcription factor KLF4 and the growth factor BMP4.^103^^,^^104^ In addition, hypoxia-induced Notch signaling promotes self-renewal and metastasis in TNBC stem cells.^105^ Knockdown of Notch1 reduced the expression of the CD44^high^/CD24^low^ phenotype in TNBC cells, resulting in reduced cell proliferation, Matrigel invasion, and brain metastasis development *in vivo*.^106^ Furthermore, hN1-NRR/Fc, an anti-Notch antibody, reduces mammosphere formation by decreasing the CD44^high^/CD24^low^ cell population in TNBC cells, resulting in a lower tumor incidence after reimplantation and late tumor recurrence.^107^ Grudzien et al.^108^ used three structurally unique gamma-secretase inhibitors, Z-Leu-Leu-Nle-CHO, LY-411575, and MRK003, as well as a Notch-1-Fc or Notch1 siRNA, to support that Notch signaling is critical for CSC maintenance in TNBC. This section discusses drugs that suppress Notch signaling to decrease the number of TNBC stem cells.

#### Celastrol

Celastrol is a terpenoid derived from *Tripterygium wilfordii*, also known as Thunder God Vine in traditional Chinese medicine. Celastrol treatment inhibited mammosphere formation in TNBC and the expressions of DCLK1, ALDH1, and CD133 and inhibited Notch1 activation. Notch downstream target proteins HES-1 and HEY-1 were also downregulated.^109^

#### Triptolide

Triptolide is also a terpene produced from *T. wilfordii*. Mammosphere development and the expression of the CSC markers DCLK1, ALDH1, and CD133 were decreased in triptolide-treated TNBC cells. These effects are associated with the inhibition of Notch1 signaling.^109^

#### DAPT

DAPT is a γ-secretase inhibitor that interferes with Notch signaling. In 231-BR cells, the brain metastatic variant of MDA-MB-231 TNBC cells, the CD44^high^/CD24^low^ phenotype was reduced by DAPT treatment, and DAPT-treated mice presented reduced metastasis development.^106^

#### Psoralidin

Psoralidin is an important bioactive chemical produced from *Psoralea corylifolia* seeds. Psoralidin (25 mg/kg) reduced the growth of ALDH^+^ and ALDH^−^ tumors by acting as a gamma secretase inhibitor. Furthermore, psoralidin-mediated suppression of Notch1 inhibited EMT activation in ALDH^+^ and ALDH^−^ cancer cells.^110^

These findings reinforce that the Notch signaling pathway plays a crucial role in maintaining the stem cell properties of TNBC. Although these preclinical results are promising, clinical trials are needed to validate the efficacy and safety of these molecules in TNBC patients.

### Hippo signaling pathway

**Figure 5:**
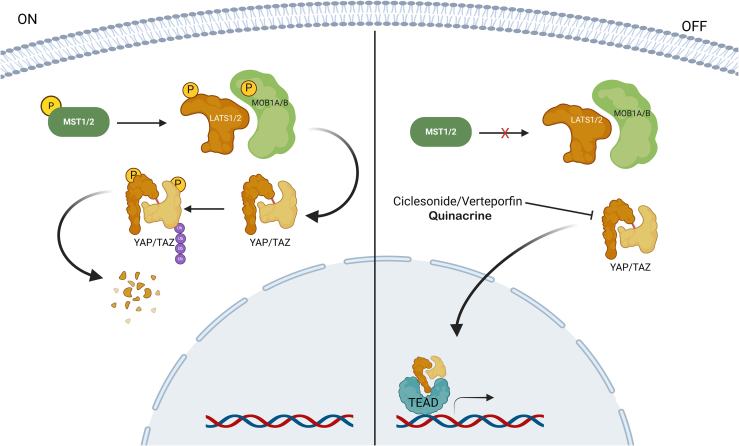
Hippo cell signaling pathway When activated, the Hippo pathway phosphorylates the coactivators YAP/TAZ, preventing their entry into the nucleus and limiting their ability to control cells. When inactive, YAP/TAZ translocate to the nucleus and activate genes that promote growth, survival, and tissue regeneration.

The Hippo pathway comprises a kinase cascade (mammalian sterile 20-like kinase, MST; and large tumor suppressor, LATS) and a downstream transcriptional module (yes-associated protein, YAP; and transcriptional coactivator with PDZ-binding motif, TAZ). MST1/2 phosphorylates and activates the downstream kinases LATS1/2 and their scaffold MOB kinase activators 1A and 1B (MOB1A/B), causing YAP and TAZ to be phosphorylated and translocated to the cytoplasm, where they are degraded by the ubiquitin proteasome pathway. When the Hippo pathway is switched off, dephosphorylated YAP and TAZ are delivered to the nucleus and activate gene expression via transcriptional-enhancer-associated domain (TEAD) transcription factors (Figure 5).^111^^,^^112^

The Hippo TAZ transducer endows breast cancer cells with CSC-like properties.^113^ Although YAP hyperactivation in mammary epithelia does not cause hyperplasia, it does result in abnormalities in terminal differentiation. Furthermore, in a PyMT mouse model of YAP-deficient breast cancer, the incidence of lung metastases decreased.^114^ YAP has been shown to control the transcription of stem cell signature genes, promote tumorsphere development, and promote chemoresistance in TNBC cells.^115^^,^^116^ Hippo/YAP signaling modulators that target TNBC stem cells are discussed in this section.

#### Ciclesonide

Ciclesonide is a clinically approved glucocorticoid used to treat asthma and allergic rhinitis. Ciclesonide reduced TNBC cell growth and promoted apoptosis, resulting in fewer CD44^+^/CD24^−^ and ALDH^+^ cancer cells. In ciclesonide-treated TNBC cells, there was ubiquitination-dependent degradation of the glucocorticoid receptor and a decrease in the protein level of YAP. Furthermore, ciclesonide at 10 mg/kg inhibited tumor growth in MDA-MB-231 tumor-bearing nude mice.^117^

#### Verteporfin

Verteporfin is a benzoporphyrin derivative that acts as a photosensitizing agent in photodynamic therapy, being activated by light to induce selective cytotoxic effects. The combination of the clinically approved drugs paclitaxel, verteporfin, and combretastatin in polymer-lipid hybrid nanoparticles was assessed for the treatment of TNBC. In the treatment of macular degeneration, verteporfin has been found to inhibit the Hippo/YAP pathway. This nanoparticle effectively reduced the viability and migration of MDA-MB-231 cells. Additionally, paclitaxel-induced CSC enrichment is significantly diminished by this nanoparticles, which is due in part to the inhibition of Hippo/YAP signaling.^118^

The synergistic effect of verteporfin and doxorubicin treatment on the viability of the TNBC cell line MDA-MB-231 was assessed via the MTT assay. After 24 h of pretreatment with verteporfin, MDA-MB-231 cells were effectively sensitized to doxorubicin, which significantly reduced survival.^119^

#### Quinacrine

Quinacrine, also known as mepacrine, Atabrine, or Atebrin, is a derivative of 9-aminoacridine that inhibits TNBC stem cells.^120^ Quinacrine treatment also significantly decreased the expression of the YAP gene in MDA-MB-231 cells, indicating suppression of Hippo signaling.^121^

These data support the crucial importance of the Hippo/YAP pathway in the regulation of CSCs in TNBC. Modulation of this system offers promising therapeutic approaches to combat treatment resistance and metastasis associated with these cells. In any case, further clinical trials are needed to determine the efficacy and safety of these drugs in patients with TNBC.

### TGF-β signaling pathway

**Figure 6:**
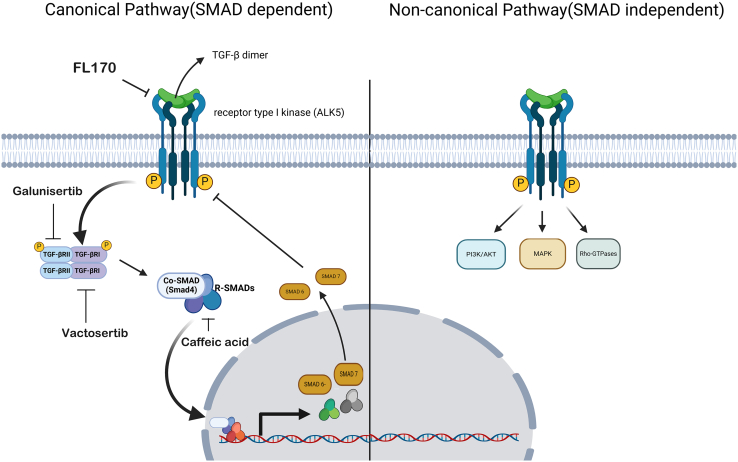
TGF-β signaling pathway TGF-β binds to its active receptors, such as SMAD proteins, which form a complex and translocate to the nucleus, regulating the expression of genes involved in specificity, differentiation, apoptosis, and the immune response. In noncanonical activation, the signal is transmitted via other pathways, such as the MAPK, PI3K/AKT, and Rho-type GTPase pathways, influencing processes such as migration, cell survival, and tissue remodeling.

The TGF-β signaling pathway is a complex cellular signaling network consisting of two branches: a canonical pathway transduced by SMADs and a noncanonical pathway independent of SMAD proteins (Figure 6). These branches activate different target genes and often have competing physiological effects.^122^^,^^123^

In canonical signaling, the interaction between TGF-β ligands initiates the formation of a heterotetrameric active receptor complex consisting of a TGF-β dimer alongside homodimers of TGF-β receptor type II (TGF-βRII) and TGF-β receptor type I (TGF-βRI). This interaction results in the phosphorylation of TGF-βRI by TGF-βRII. TGF-βRI subsequently phosphorylates Smad-R proteins, specifically Smad1, Smad2, Smad3, Smad5, and Smad8, which then associate with co-Smad, known as Smad4. This complex translocates to the nucleus, where it collaborates with other DNA-binding transcription factors to regulate the transcription of target genes. Furthermore, inhibitory SMADs, such as SMADs 6 and 7, function as I-SMADs that can attenuate this signaling process.^122^^,^^123^

In cancer development and progression, TGF-β suppresses tumors in the early stages but promotes them later in cancer progression.^122^^,^^123^ In both *in situ* and invasive human breast carcinomas, loss of TGF-βRII expression is associated with high tumor grade.^124^ This section discusses TGF-β signaling inhibitors that act on TNBC stem cells.

#### Galunisertib

Galunisertib, also known as LY2157299, is a TGF-βRII-neutralizing antibody. Galunisertib inhibited paclitaxel-induced IL-8 transcription and CSC proliferation in TNBC cells. Furthermore, galunisertib treatment of TNBC xenografts inhibited tumor regrowth after paclitaxel therapy.^125^

#### Vactosertib

Vactosertib, also known as EW-7197, is an orally bioavailable inhibitor of TGF-βRI kinase (also known as ALK5). Treatment with vactosertib inhibited paclitaxel-induced EMT and CSC features, such as mammosphere development, ALDH activity, the CD44^+^/CD24^-^subset, and pluripotency regulators (NANOG, MYC, Klf4, OCT4, and SOX2). Furthermore, vactosertib enhances the therapeutic impact of paclitaxel by reducing lung metastases and improving *in vivo* survival time.^126^ Vactosertib also suppressed radiation-induced EMT and CSC features in TNBC cells through reducing reactive oxygen species (ROS) stress. Furthermore, vactosertib in combination with radiation has a strong antimetastatic effect, suppressing lung metastasis *in vivo*.^127^

#### ZL170

ZL170 is a natural component of *Periplaneta americana*. ZL170 reduced TGF-β and bone morphogenetic protein (BMP) receptor kinase activity and Smad activation in TNBC cells, decreasing Snail and Slug expression and suppressing the EMT process. Furthermore, ZL170 therapy reduced NANOG and SOX2 expression, decreased CD49f and CD44 levels, and decreased the percentage of ALDH1. ZL170 at 80 mg/kg inhibited TNBC osteolytic bone metastasis and xenograft tumor growth, as well as primary tumor growth and lung metastases, in PyMT transgenic mice.^128^

#### Caffeic acid

Caffeic acid is an active ingredient in propolis phenolic extract. Although caffeic acid had a minor effect on cancer cell viability, it reduced the expression of CD44, EpCAM, and/or ALDH1, as well as OCT4, BMI-1, and Lin-28B, in TNBC cells. Furthermore, caffeic acid suppressed SMAD2 through the demethylation of MiR-148a.^129^

These data corroborate that the TGF-β pathway is essential for the maintenance of TNBC stem cell characteristics, such as self-renewal, chemotherapy resistance, and metastatic capacity. The mentioned molecules act at different points in this pathway. Despite the promising preclinical results, it is necessary to advance clinical trials to evaluate the efficacy and safety of these molecules in humans.

### JAK/STAT signaling pathway

**Figure 7:**
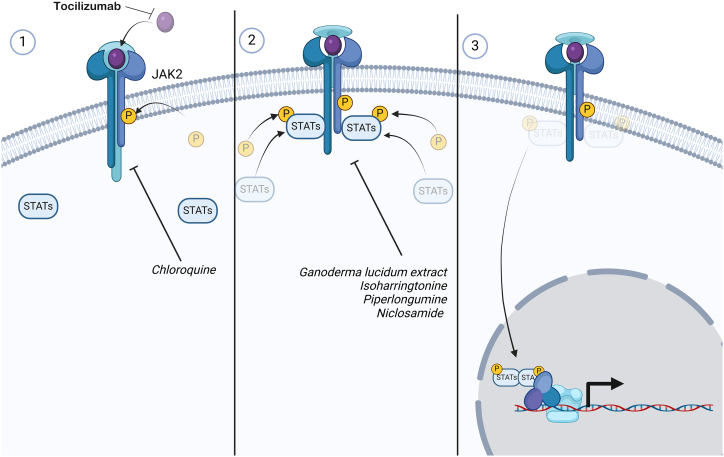
JAK/STAT cell signaling pathway The binding of cytokines to their receptors activates JAK kinases, which phosphorylate STAT transcription factors. After being phosphorylated, STATs dimerize and migrate to the nucleus, where they regulate the expression of genes related to intolerance, differentiation, and the immune response.

The JAK/STAT signaling pathway is associated with many cytokines and growth factors and controls a wide range of biological activities. It consists of ligand-receptor complexes, JAKs, and STATs (Figure 7). The JAK family has four members: JAK1, JAK2, JAK3, and TYK2. The STAT family has seven members: STAT1, STAT2, STAT3, STAT4, STAT5a, STAT5b, and STAT6.^130^^,^^131^

The activation of the signaling pathway is initiated by the interaction of the extracellular ligand with its receptor, JAK, which induces conformational changes in the receptors. These changes facilitate the phosphorylation of associated intracellular JAKs. Once transphosphorylated, these JAKs phosphorylate a variety of downstream substrates, including receptor and STAT proteins. Phosphorylated STATs subsequently translocate to the nucleus, where they form dimers or more intricate oligomeric structures that bind to specific enhancer regions within target genes, thereby modulating their transcriptional activity.^130^^,^^131^

The JAK/STAT signaling pathway is one of the major dysregulated pathways in breast cancer and plays an important role in the regulation of CSCs.^130^ Marotta et al.^132^ reported that the IL-6/JAK2/STAT3 pathway was more active in CD44^+^CD24^−^ TNBC cells than in non-CSCs and that JAK2 inhibition reduced their number and xenograft growth. Furthermore, Thiagarajan et al.^133^ demonstrated that leptin may regulate the proliferation and development of TNBC stem cells by activating JAK/STAT signaling. Similarly, Liu et al.^134^ reported that HN1L promotes TNBC stem cells via the LEPR-STAT3 pathway. Recently, Wu et al.^135^ reported that the circKIF4A-miR-637-STAT3 axis stimulates brain metastasis in TNBC. Drugs that suppress TNBC stem cells through JAK/STAT signaling are discussed in this section.

#### Ganoderma lucidum extract

Rios-Fuller et al.^136^ demonstrated that the extract of *Ganoderma lucidum*, a medicinal mushroom with anticancer activity, targets breast CSCs *in vitro* and TNBC tumors in animal models via downregulation of the STAT3 pathway. *G. lucidum* extract inhibited the growth of TNBC cells and decreased the expression of total and phosphorylated STAT3. These effects include a reduction in OCT4, NANOG, and SOX2; a decrease in the breast CSC population due to the depletion of ALDH1 and CD44^+^/CD24^−^; deformation of mammospheres; and tumor shrinkage in mouse models.^136^

A retrospective clinical trial of TNBC patients who received *G. lucidum* spore powder revealed that *G. lucidum* may improve overall survival and disease-free survival in individuals with early-stage TNBC. Patients who consumed *G. lucidum* had a better overall survival rate, especially those with stages II and III disease.^137^ Furthermore, a pilot clinical study in patients with ER-positive breast cancer revealed that *G. lucidum* spore powder can reduce fatigue and improve quality of life in breast cancer patients receiving endocrine therapy without causing severe side effects.^138^

#### Isoharringtonine

Isoharringtonine is a natural analog of homoharringtonine and is extracted from *Cephalotaxus harringtonia*. In a panel of TNBC cell lines, isoharringtonine inhibited the proliferation and migration of cells. More importantly, isoharringtonine reduced the proportion of CD44^+^/CD24^−^ cells in a dose-dependent manner and inhibited mammosphere formation, indicating that isoharringtonine has anti-TNBC stem cells effects. Additionally, isoharringtonine suppressed the expression of total and phospho-STAT3 and NANOG. Taken together, these results indicate that isoharringtonine targets TNBC stem cells through the inhibition of the STAT3 pathway.^139^

#### Piperlongumine

Piperlongumine, also known as piplartine, is an alkaloid amide from the *Piper* genus with multiple pharmacological properties, including antitumor activity against a wide variety of tumors.^140^^,^^141^^,^^142^ A PLGA-based nanoformulation for piperlongumine inhibited ALDH expression, self-renewal, chemoresistance, and EMT in mammospheres formed from TNBC cells by inhibiting STAT3.^143^ Interestingly, piperlongumine was also able to reduce the leukemic stem cell population of acute myeloid leukemia cells.^144^

#### Chloroquine

Chloroquine is an antimalarial drug with anticancer potential that acts by inhibiting autophagy.^145^ Chloroquine sensitized TNBC cells to paclitaxel by inhibiting autophagy and reducing the CD44^+^/CD24^−/low^ CSC population by reducing JAK2 and DNA methyltransferase 1 expression.^146^

In a phase 2 clinical trial, the efficacy and safety of chloroquine combined with taxanes in patients with anthracycline-resistant advanced or metastatic breast cancer were evaluated. Every 3 weeks, 250 mg of chloroquine was administered orally together with docetaxel, paclitaxel, nab-paclitaxel, or ixabepilone, and 31 patients, including eight with TNBC, were evaluated for response. The objective response rate was 45.16%, exceeding the projected objective response rate of 30%.^147^

#### Tocilizumab

Tocilizumab is a clinically approved humanized anti-IL-6R neutralizing antibody that inhibits IL-6 signaling by competing with soluble and membrane-bound IL-6R. Alraouji et al.^148^ reported that tocilizumab inhibited the IL-6/STAT3/NF-κB autocrine positive feedback loop in TNBC cells. Tocilizumab treatment also inhibited the Wnt/β-catenin pathway, EMT, and stemness-related features in TNBC cells. This included reduced levels of CD44 and increased levels of CD24, as well as downregulation of NANOG, OCT4, SOX2, and KLF4 expression. Furthermore, tocilizumab enhances the effects of cisplatin in both *in vitro* and *in vivo* models.^148^

#### Niclosamide

Niclosamide is an oral anthelmintic drug used to treat tapeworm infestations such as diphyllobothriasis, hymenolepiasis, and taeniasis. Niclosamide and its hyaluronic acid nanoconjugate inhibited the CD44^high^/CD24^low^ population of TNBC cells while decreasing the proportion of side population and mammosphere development. Furthermore, niclosamide and its hyaluronic acid nanoconjugate efficiently downregulated two activated STAT3 forms, pY705 and pS727.^149^

These data support the idea that the JAK/STAT pathway plays a crucial role in maintaining TNBC stem cell properties, including self-renewal, chemotherapy resistance, and metastatic capacity. Interestingly, these molecules act at different points in this pathway and have demonstrated anti-TNBC potential in preclinical studies. Therefore, future clinical trials are needed to determine the efficacy and safety of these compounds in TNBC patients.

### PI3K/AKT/mTOR signaling pathway

**Figure 8:**
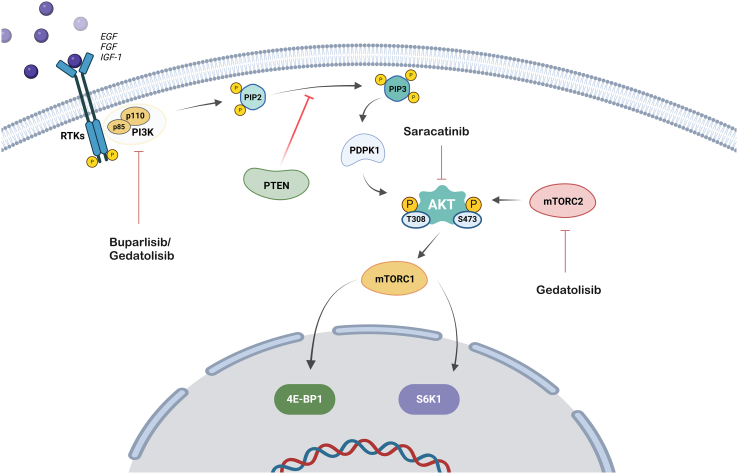
PI3K/AKT/mTOR cell signaling pathway Activation of the membrane receptor stimulates PI3K, which generates signaling molecules that activate AKT. AKT promotes cell survival and growth by activating the mTOR pathway, which regulates protein synthesis and cellular metabolism.

The phosphatidylinositol 3-kinase (PI3K)/AKT/mammalian target of rapamycin (mTOR) signaling pathway is known to play an important role in tumor cell growth and proliferation in response to food availability, hormones, and growth factor stimulation. The PI3K heterodimer, which belongs to class IA of PI3Ks, plays a critical role in this pathway. The heterodimer is composed of two subunits, with the regulatory subunit (p85) controlling whether the catalytic subunit (p110) is activated in response to upstream stimulation by growth factor receptor tyrosine kinases. PI3Ks phosphorylate phosphatidylinositol 4,5-bisphosphate (PIP2) to phosphatidylinositol 3,4,4-trisphosphate (PIP3), which then phosphorylates AKT, a serine/threonine kinase (Figure 8). Phosphatase and tensin homolog deleted on chromosome 10 (PTEN) functions in the opposite direction by dephosphorylating PIP3 to PIP2.^150^^,^^151^^,^^152^

mTOR is a serine/threonine protein kinase that exists downstream of PI3K and AKT. The term mTOR refers to two separate complexes, mTORC1 and mTORC2, which operate in different ways. Rapamycin and its equivalents target mTORC1, whereas mTORC2 is blocked by these drugs at sufficient levels, affecting cellular metabolism and cancer cell proliferation. AKT activates the mTORC1 protein. mTORC1 influences cellular metabolism and promotes anabolic cell development by interacting with 40S ribosomal protein S6 kinase 1 (S6K1) and eukaryotic initiation factor 4E-binding protein (4EBP1).^150^^,^^151^^,^^152^^,^^153^ mTORC1 also induces docetaxel resistance and liver metastases in TNBC cells.^154^

Several research studies have revealed the role of this pathway in the maintenance of CSCs. Sulaiman et al.^155^ reported that TNBC stem cells have higher levels of mTORC1 than non-stem TNBC cells do. Furthermore, Bai et al.^156^ demonstrated that HIF-2α promotes TNBC stem cells by regulating CD44 via PI3K/AKT/mTOR signaling. Britschgi et al.,^157^ on the other hand, demonstrated that inhibition of PI3K/AKT/mTOR signaling increased the activation of JAK2/STAT5 signaling in a positive feedback loop, reducing the efficacy of PI3K/mTOR inhibition, implying that TNBC cells should be controlled through a combination of PI3K/mTOR and JAK2/STAT5 pathway inhibition. This section discusses drugs that decrease TNBC stem cells through PI3K/AKT/mTOR signaling.

#### Buparlisib

Buparlisib, also known as NVP-BKM120 or BKM120, is an orally bioavailable new generation of PI3K-specific inhibitor. Yu et al.^158^ reported that buparlisib reduces the proliferation of TNBC cells, including CSCs, through the inhibition of the PI3K/AKT/mTOR signaling pathway. Furthermore, buparlisib synergized with RAD001 (mTOR inhibitor/also known as everolimus) to decrease the proliferation of TNBC cells *in vitro* and *in vivo*.

A phase 1 dose-escalation clinical trial in patients with advanced solid tumors indicated a maximum safe and well-tolerated dose of 100 mg/day for buparlisib, and a patient with TNBC demonstrated a confirmed partial response.^159^ In a phase 2 clinical trial in patients with TNBC, 50 participants were enrolled and treated with buparlisib at a starting dose of 100 mg daily. However, no confirmed objective response was detected, indicating that PI3K pathway blockade alone may not be sufficient as a therapeutic strategy for TNBC.^160^

#### Saracatinib

Saracatinib, also known as AZD0530, inhibits Src, a membrane-associated nonreceptor tyrosine kinase. Saracatinib has synergistic antiproliferative effects with gemcitabine and partially overcomes gemcitabine resistance in TBNC cells by inhibiting migratory and stem cell characteristics via the AKT/c-Jun pathway.^161^ On the other hand, a phase 2 clinical trial of saracatinib for the treatment of patients with hormone receptor-negative metastatic breast cancer demonstrated that this agent alone has no significant activity.^162^

#### Gedatolisib

Gedatolisib, also known as PF-04691502, is a dual inhibitor that targets mTOR and PI3K. In patient-derived TNBC cells, gedatolisib reduces mammosphere development while preferentially targeting the CSC component.^163^ In an open-label phase 1B clinical trial, combination therapy with gedatolisib demonstrated adequate tolerability and clinical efficacy at the recommended phase 2 dose in patients with TNBC.^164^

Overall, the PI3K/AKT/mTOR pathway is critical for maintaining stem cell characteristics in TNBC. However, more clinical trials are needed to determine the efficacy and safety of these compounds in TNBC patients. In particular, the interaction of PI3K/AKT/mTOR with JAK2/STAT5 signaling should be considered to effectively suppress cancer cells.

## Conclusions and future perspectives

In recent years, substantial progress has been made in understanding TNBC stem cells, notably, the discovery that some cell signaling pathways are dysregulated in this subset of cancer cells and that they regulate a wide range of CSC behaviors, including proliferation, resistance, and metastasis, which has proven critical in the development of therapeutic approaches targeting CSCs. These approaches include the use of therapeutic agents to block signaling pathways required for CSC maintenance and self-renewal. This study examined inhibitors of the HH, NF-κB, Wnt, Notch, Hippo, TGF-β, JAK/STAT, and PI3K/AKT/mTOR signaling pathways as potential treatment approaches to eliminate TNBC stem cells. A total of 45 cell signaling inhibitors were found to eliminate TNBC stem cells in culture via cell lines or primary cells. These data are summarized in Table S2.

Although promising results have been reported in preclinical models, only apatinib, buparlisib, galunisertib, gedatolisib, resveratrol, and tocilizumab have been examined in clinical studies, as registered at www.clinicaltrials.gov (Table S3). Twenty-one of them were related to apatinib, six to buparlisib, one to galunisertib, three to gedatolisib, one to resveratrol and one tocilizumab. Seven phase 1 and 2 clinical trials have revealed that apatinib can improve the treatment of patients with TNBC,^88^^,^^89^^,^^90^^,^^91^^,^^92^^,^^93^^,^^94^ indicating that this molecule should be tested in phase 3 clinical trials. Interestingly, there is an ongoing phase 3 clinical trial with this drug in TNBC patients (NCT06889688), although one phase 3 clinical trial conducted with apatinib has been terminated owing to an adjustment in the sponsor’s R&D strategy (NCT04335006). These data suggest that apatinib may be approved for the treatment of TNBC soon.

A phase 1 clinical trial with buparlisib^159^ and gedatolisib^164^ in patients with TNBC demonstrated a safe and partial response to these drugs. However, data from phase 2 clinical trials with buparlisib demonstrated no benefit in patients with TNBC when this therapeutic PI3K inhibitor was administered alone.^160^ Indeed, as mentioned previously, in experimental investigations, the isolated blockade of the PI3K/AKT/mTOR pathway resulted in the activation of the JAK2/STAT5 pathway, demonstrating that these signaling pathways are interconnected.^157^ Crosstalk between biological signaling pathways, including Wnt/β-catenin with NF-κB signaling,^165^ Wnt/β-catenin with HH signaling,^166^ TGF-β with JAK/STAT3 signaling,^167^ and Notch1 with NF-κB signaling,^168^ is common. Therefore, to achieve effective suppression of this communication network, more than one signaling pathway must be blocked.

Results from the phase 1 clinical trial of galunisertib in patients with TNBC (NCT02672475) have not been reported, but it has been studied in several clinical trials for various cancer types, both as monotherapy and in combination with other treatments with preliminary efficacy.^169^^,^^170^ In the case of the only clinical trial registered at clinicaltrials.gov with resveratrol in patients with TNBC (NCT04266353), this trial was withdrawn because of COVID-19.

Although not detected in our clinicaltrials.gov search, the combination of curcumin with paclitaxel in a published clinical study outperformed the paclitaxel-placebo combination in 150 women with advanced and metastatic breast cancer (including seven patients with TNBC).^49^ Similarly, sonidegib was evaluated in combination with docetaxel in patients with advanced TNBC in a phase 1b study, and the combination showed anticancer activity in 3 of 10 patients with detectable disease.^64^ A retrospective clinical study of TNBC patients who received *G. lucidum* spore powder revealed that *G. lucidum* may improve overall survival and disease-free survival in TNBC patients.^137^ Chloroquine combined with taxanes in patients with anthracycline-resistant advanced or metastatic breast cancer, including eight patients with TNBC, has also been shown to be an improved treatment.^147^

Although CSC-targeted drugs have the potential to improve cancer treatment, several issues need to be considered. CSCs are particularly heterogeneous, which increases the difficulty of eradicating this subpopulation of cells since CSCs can adjust to block a single specific pathway by activating compensatory pathways, which are interconnected, as mentioned above. Furthermore, some signaling pathways play physiological roles in normal stem cells,^171^ resulting in unfavorable outcomes.

Additionally, the tumor microenvironment can alter tumor drug sensitivity, increasing treatment difficulty. The tumor microenvironment contains vascular niches, hypoxia, immune cells, fibroblasts, mesenchymal stem cells, the extracellular matrix, and exosomes. Hypoxia activates hypoxia-inducible factors (HIFs), which maintain the indistinguishable state of CSCs.^104^ Tumor-associated macrophages promote the formation and survival of CSCs through pathways such as the TGF-β and STAT3 pathways. Tumor-associated macrophages promote the transformation of non-CSCs into CSCs through factors such as IL-6, TGF-β, and metalloproteinases.^172^ Mesenchymal stem cells interact with CSCs to promote cell growth and metastasis, including cell fusion.^173^ The extracellular matrix regulates the plasticity and resistance of CSCs through signals such as Wnt and YAP/TAZ.^174^ Exosomes transport molecules that affect the formation, survival, and resistance of CSCs. They also promote the conversion of non-CSCs into CSCs.^175^ As a result, the tumor microenvironment is also essential for maintaining the malignant properties of CSCs and serves as a significant therapeutic target. Therefore, for signaling pathway inhibitors to improve the treatment of cancer patients, all these issues need to be considered.

These findings suggest that more preclinical and clinical trials with cell signaling inhibitors targeting TNBC stem cells should be conducted to improve patient treatment in the future. This involves the design of dual inhibitors that target several pathways, drugs combinations, the study of the role of noncoding RNAs in CSC signaling, the development of nanotechnology for targeted delivery, and the use of artificial intelligence to repurpose drugs in TNBC.

## Acknowledgments

F.P.O., M.L.N., A.F.C.G., and R.B.D. received personal scholarship from 10.13039/501100002322Coordenação de Aperfeiçoamento de Pessoal de Nível Superior (code 001, CAPES, Brazil), and D.P.B. received personal scholarship from 10.13039/501100003593Conselho Nacional de Desenvolvimento Científico e Tecnológico (CNPq, Brazil).

## Author contributions

M.L.N., F.P.O., and A.F.C.G. drafted the article content, and R.B.D. and D.P.B. planned this review and reviewed the whole text. All the authors read and approved the final manuscript.

## Declaration of interests

The authors declare that there are no conflicts of interest.

## Supplemental information

Document S1. Tables S1–S3

Document S2. Article plus supplemental information
