# Supplementary material for: Translational drugs targeting cancer stem cells in triple-negative breast cancer
Source: Mol Ther Oncol. 2025 Jun 13;33(3):201008. doi: 10.1016/j.omton.2025.201008 (PMC12269295; doi:10.1016/j.omton.2025.201008)
Supplement: Document S1. Tables S1–S3 [file mmc1.pdf]

OMTON, Volume *33*

Supplemental information

Translational drugs targeting cancer stem cells in triple-negative breast cancer

Felipe P. de Oliveira, Mateus L. Nogueira, Alexandre F.C. Galvão, Rosane B. Dias, and Daniel P. Bezerra

Table S1. Major TNBC stem cell markers

Biomarker	Function	Relevance in the TNBC	References
$CD44^{+}/CD24^{-}$	Classical CSC markers	Highly expressed in TNBC stem cells; associated with metastasis and therapeutic resistance	12
ALDH1 (Aldehyde dehydrogenase 1)	Cellular detoxification enzyme	High activity correlates with self-renewal and aggressiveness of TNBC	13
EpCAM (CD326)	Epithelial adhesion molecule	Indicator of CSCs in several cancer subtypes, including TNBC	12
CD133 (Prominin-1)	Membrane Glycoprotein	Associated with Tumorigenesis and Chemoresistance in TNBC	14
SOX2, OCT4, NANOG	Pluripotent Transcription	Factors Regulate CSC Self-Renewal and Pluripotency in TNBC	17,18
LGR5	Surface receptor linked to the Wnt pathway	Implicated in the maintenance of CSCs and progression of TNBC	19
ABCG2	ATP-binding cassette subfamily G member 2	Involved in the resistance of CSCs	15

Table S2. Cell signaling inhibitors targeting TNBC stem cells

Drug	Source	Target	References
6-Methoxymellein	Natural product	NF-κB signaling	75
Apatinib	Synthetic	Wnt signaling	87
Buparlisib	Synthetic	PI3K/AKT/mTOR signaling	158
Caffeic acid	Natural product	TGF-β signaling	128
Caffeic acid phenethyl ester	Natural product	NF-κB signaling	74
Celastrol	Natural product	Notch signaling	108
Chloroquine	Synthetic	JAK/STAT signaling	146
Ciclesonide	Synthetic	Hippo signaling	116
Curcumin	Natural product	Wnt signaling<br>HH signaling	47,48
CWP232228	Synthetic	Wnt signaling	95
DAPT	Synthetic	Notch signaling	105
Diallyl trisulfide	Natural product	Wnt signaling	96
Diosgenin	Natural product	Wnt signaling	97
Disulfiram	Synthetic	NF-κB signaling	76
Eugenol	Natural product	NF-κB signaling	73
Galunisertib	Synthetic	TGF-β signaling	124
Ganoderma lucidum extract	Natural product	JAK/STAT signaling	136
Gedatolisib	Synthetic	PI3K/AKT/mTOR signaling	163
GANT61	Synthetic	HH signaling	37
Gomisin M2	Natural product	Wnt signaling	84
Hydroxytyrosol	Natural product	Wnt signaling<br>TGF-β signaling	98
ICG-001	Synthetic	Wnt signaling	85

Isoharringtonine	Natural product	JAK/STAT signaling	139
Machilin D	Natural product	NF-κB signaling	78
Niclosamide	Synthetic	JAK/STAT signaling	149
Nitidine chloride	Natural product	HH signaling	65
Physalin A	Natural product	HH signaling<br>Hippo signaling	66
Piperlongumine	Natural product	JAK/STAT signaling	143
Psoralidin	Natural product	Notch signaling	109
Pterostilbene	Natural product	NF-κB signaling	79
Quinacrine	Synthetic	Hippo signaling	120
Resveratrol	Natural product	Wnt signaling	99
Saracatinib	Synthetic	PI3K/AKT/mTOR signaling	161
SHH002-hu1	Biotechnological	Wnt signaling	86
Sonidegib	Synthetic	HH signaling	42
Sulconazole	Synthetic	NF-κB signaling	80
Thiostrepton	Natural product	HH signaling	59
Tocilizumab	Biotechnological	JAK/STAT signaling<br>NF-κB signaling<br>Wnt signaling	148
Trans-[Pt(II)Cl_2(dmso)(GANT61)]	Synthetic	HH signaling	62
Triptolide	Natural product	Notch signaling	108
Ursolic acid	Natural product	Wnt signaling	100
Vactosertib	Synthetic	TGF-β signaling	125,126
Verteporfin	Synthetic	Hippo signaling	117,118
XAV-939	Synthetic	Wnt signaling	74
ZL170	Natural product	TGF-β signaling	127

Table S3. Cell signaling inhibitors that target TNBC stem cells and are being evaluated in clinical trials as anti-TNBC agents*

ClinicalTrials.gov ID	Tile	Conditions	Intervention/ Treatment	Phase	Study Start	Current Status
NCT03805399	Precision treatment of refractory triple negative breast cancer based on molecular subtyping --FUSCC-TNBC- umbrella trial	Triple-negative breast cancer	Drug: Pyrotinib with Capecitabine<br>Drug: AR inhibitor combined with everolimus (B1) or CDK4/6 inhibitor (B2), or EZH2 inhibitor (B4)<br>Drug: anti PD-1 with nab-paclitaxel<br>Drug: PARP inhibitor included therapy<br>Drug: BLIS with anti-VEGFR included therapy<br>Drug: MES with anti-VEGFR included therapy<br>Drug: mTOR inhibitor with nab-paclitaxel	Phase 1 Phase 2	2018-10-18	Unknown status
NCT05447702	A single-arm, prospective phase II study of camrelizumab plus apatinib and chemotherapy as neoadjuvant therapy for triple negative breast cancer (TNBC)	Triple negative breast cancer	Drug: Camrelizumab<br>Drug: Apatinib<br>Drug: Nab-paclitaxel<br>Drug: Epirubicin	Phase 2	2022-11-01	Recruiting

			Drug: Cyclophosphamide			
NCT05556200	A Phase II Trial of Camrelizumab in Combination With Apatinib for Neoadjuvant Treatment of Early-stage TNBC With a High Proportion of TILs	Breast Cancer<br>Triple-Negative Breast Cancer	Drug: Anti-PD-1 monoclonal antibody<br>Drug: VEGFR2 Tyrosine Kinase Inhibitor	Phase 2	2022-12-01	Recruiting
NCT03945604	A Phase Ib, Open-labeled, Multicenter, Dose-exploring Trial of SHR-1210 (Anti-PD-1 Antibody) in Combination With Apatinib and Fluzoparib in Subjects With Recurrent and Metastatic Triple Negative Breast Cancer	Triple Negative Breast Cancer	Drug: SHR-1210 + Apatinib +Fluzoparib	Phase 1	2019-06-04	Completed
NCT05192798	A Prospective, Randomized, Open Label Clinical Study Evaluating Efficacy and Safety of Albumin-Bound Paclitaxel Combined With Antiangiogenic Agents in First-line Treatment of Relapsed or Metastatic Triple Negative Breast Cancer	Triple-negative Breast Cancer	Drug: Albumin-Bound Paclitaxel<br>Drug: Apatinib Mesylate<br>Drug: Bevacizumab	Phase 2	2022-01-14	Recruiting
NCT03394287	A Phase II, Open-labeled, Randomized, Noncomparative, Two-arms Investigator-initiated Clinical Trial of SHR-1210 (Anti-PD-1 Antibody) in Combination With Apatinib in Subjects With Advanced Triple Negative Breast Cancer	Breast Cancer	Drug: SHR-1210<br>Drug: Apatinib	Phase 2	2018-01-10	Completed
NCT04303741	An Open-labeled, Single-arm, Investigator-initiated Phase II Trial of Camrelizumab (Anti-PD-1 Antibody) in Combination With Apatinib	Breast Cancer	Drug: Camrelizumab<br>Drug: Apatinib<br>Drug: Eribulin	Phase 2	2020-03-25	Unknown status

	and Eribulin in Patients With Advanced Triple-Negative Breast Cancer					
NCT05582499	Fudan University Shanghai Cancer Center Breast Cancer Precision Platform Series Study- Neoadjuvant Therapy (FASCINATE-N)	Breast Neoplasm<br>Breast Cancer<br>Breast Tumors<br>Triple-Negative Breast Cancer (TNBC)<br>HER2-positive Breast Cancer<br>HER2-negative Breast Cancer<br>Hormone Receptor Positive Tumor<br>Hormone Receptor Negative Tu mor<br>Early stage Breast Cancer<br>Locally Advanced Breast Cancer	Drug: Dalpiciclib<br>Drug: Pyrotinib<br>Drug: SHR-A1811<br>Drug: SHR-1316<br>Drug: Camrelizumab<br>Drug: SHR-A1921<br>Drug: Pertuzumab<br>Drug: Trastuzumab<br>Drug: Goserelin<br>Drug: Letrozole<br>Drug: Nab paclitaxel<br>Drug: Carboplatin<br>Drug: Epirubicin<br>Drug: Cyclophosphamide<br>Drug: Fluzoparib<br>Drug: Apatinib<br>Drug: Famitinib	Phase 2	2022-11-01	Recruiting
NCT03243838	Low-dose Apatinib Combined with Neoadjuvant Chemotherapy in the Treatment of Early Triple Negative Breast Cancer (LANCET): A Multicenter, Single-arm, Phase II Trial	Triple-Negative Breast Cancer	Drug: Apatinib	Phase 2	2018-08-01	Completed
NCT03775928	Phase II Study of Compare Apatinib Plus Capecitabine Versus Capecitabine in	Triple-negative Breast Cancer	Drug: Apatinib<br>Drug: capecitabine	Phase 2	2018-12-18	Unknown status

	Maintenance Therapy for Patients with Advanced Triple-negative Breast Cancer					
NCT03348098	Tianjin Medical University Cancer Institute and Hospital	Triple Negative Breast Cancer	Drug: Apatinib<br>Drug: Paclitaxel	Phase 2	2017-09-06	Unknown status
NCT03254654	A Phase II, Single-center, Randomized Study of Vinorelbine Plus Apatinib Versus Vinorelbine as Second-Line or Third-Line Treatment in Patients With Advanced Triple-Negative Breast Cancer (NAN Trail)	Advanced Triple-Negative Breast Cancer	Drug: Vinorelbine<br>Drug: Apatinib	Phase 2	2017-08-16	Completed
NCT03932526	Combined Use of Apatinib Mesylate and Vinorelbine Versus Single Use of Vinorelbine in Recurrent or Metastatic Triple-negative Breast Cancer: a Double-blinded Randomized Controlled Clinical Trial	Triple-negative Breast Cancer	Drug: Vinorelbine + placebo<br>Drug: Vinorelbine + Apatinib	Phase 2	2019-06-24	Unknown status
NCT05019690	Apatinib Mesylate Combined with Albumin-bound Paclitaxel for Second-line Treatment of Advanced Triple Negative Breast Cancer: a Single-arm, exploratory Clinical Study	Advanced Triple Negative Breast Cancer	Drug: Apatinib Mesylate<br>Drug: Albumin-Bound Paclitaxel	Phase 1<br>Phase 2	2021-10-01	Recruiting
NCT03075462	An Open, Nonrandomized, Multicenter Phase I Study to Assess the Safety and Efficacy of Fluzoparib Given in Combination With Apatinib in Patients With Recurrent Ovarian Cancer or Triple Negative Breast Cancer	Ovarian Cancer<br>Triple Negative Breast Cancer	Drug: Fluzoparib<br>Drug: Apatinib	Phase 1	2017-03-09	Completed
NCT04335006	A Multicenter, Open-parallel, Randomized, Controlled Phase Ⅲ Study Comparing	Breast Cancer	Drug: Carelizumab<br>Drug: Nab-paclitaxel	Phase 3	2020-07-14	Terminated

	Carelizumab Plus Nab-paclitaxel and Apatinib, Carelizumab Plus Nab-paclitaxel, and Nab-paclitaxel in Patients With Unresectable Locally Advanced or Metastatic Triple Negative Breast Cancer	Triple Negative Breast Cancer	Drug: Apatinib			(Sponsor R & D Strategy Adjustment)
NCT01176669	A Single-Institutional Phase IIa Trial and A Multi-Institutional Phase IIb Trial of Apatinib in Metastatic Triple-Negative Breast Cancer	Metastatic Breast Can cer	Drug: Apatinib	Phase 2	2010-06	Completed
NCT03650738	A Prospective, One-arm Open Clinical Trial of Apatinib Combined With Albumin Paclitaxel and Carboplatin as a Neoadjuvant Therapy for the Safety and Efficacy of Triple-negative Breast Cancer	Systematic Review of the pCR Rate of Apatinib Combined With Albumin Paclitaxel and Carboplatin Regimen for Neoadjuvant Therapy of Triple-negative Breast Cance r	Drug: Apatinib	Phase 2	2018-09-01	Unknown status
NCT03735082	The Efficacy and Safety of Apatinib Combined With Paclitaxel and Carbopatin Intensive Regimen in Neoadjuvant Therapy for Locally Advanced Triple-negative Breast Cancer: Single Arm, Phase II Clinical Trail	Breast Cancer	Drug: Apatinib<br>Drug: Paclitaxel<br>Drug: Carboplatin	Phase 2	2018-11-01	Unknown status
NCT06889688	A Multicenter, Phase III, Randomized Controlled Trial Comparing Camrelizumab Plus Apatinib and Eribulin Versus Physician's	Breast Cancer Stage IV	Drug: Camrelizumab + Apatinib + Eribulin	Phase 3	2025-04-01	Active, not recruiting

	Choice Chemotherapy in the Treatment of Advanced Triple-Negative Breast Cancer		Drug: Physician's choice chemotherapy			
NCT04722718	Efficacy and Safety of Neoadjuvant Therapy with Sintilimab and Apatinib Combined Chemotherapy in Triple-negative Breast Cancer	Breast Cancer	Drug: Sintilimab + Apatinib + Albumin-Bound Paclitaxel (Nab-Paclitaxel) + Carboplatin	Phase 2	2021-02-01	Unknown status
NCT01790932	A Phase II Trial of BKM120 in Patients With Triple Negative Metastatic Breast Cancer	Breast Cancer	Drug: BKM120	Phase 2	2012-06	Completed
NCT01629615	A Phase II Trial of BKM120 (a PI3K Inhibitor) in Patients with Triple Negative Metastatic Breast Cancer	Breast Cancer	Drug: BKM120	Phase 2	2012-06	Completed
NCT02000882	Phase II Multicenter Single-arm Study of BKM120 Plus Capecitabine for Breast Cancer Patients With Brain Metastases	Brain Metastases<br>Breast Cancer<br>Metastatic Breast Cancer	Drug: BKM120<br>Drug: capecitabine<br>Drug: Trastuzumab	Phase 2	2014-05-29	Completed
NCT01623349	Phase I Study of the Oral PI3kinase Inhibitor BKM120 or BYL719 and the Oral PARP Inhibitor Olaparib in Patients With Recurrent Triple Negative Breast Cancer or High Grade Serous Ovarian Cancer	Ovarian Cancer<br>Breast Cancer	Drug: BKM120 and Olaparib<br>Drug: BYL719 and Olaparib	Phase 1	2012-09	Completed
NCT01155453	A Phase Ib, Open-label, Multicenter, Dose-escalation Study of Oral BKM120 in Combination With Oral GSK1120212 in Adult Patients With Selected Advanced Solid Tumors	Advanced and Selected Solid Tumors	Drug: BKM120<br>Drug: GSK1120212	Phase 1	2010-04	Completed

NCT01363232	A Phase Ib, Open-label, Multicenter, Dose-escalation and Expansion Study of an Orally Administered Combination of BKM120 Plus MEK162 in Adult Patients With Selected Advanced Solid Tumors	Advanced Solid Tumors Selected Solid Tumors	Drug: BKM120 + MEK162	Phase 1	2011-08	Completed
NCT02672475	A Phase Ib Trial of LY2157299 (TGFβR1 Kinase Inhibitor) With Paclitaxel in Patients with Triple Negative Metastatic Breast Cancer	Estrogen Receptor Negative HER2/Neu Negative Progesterone Receptor Negative Recurrent Breast Carci noma Stage IV Breast Cancer Triple Negative Breast Carcinoma	Drug: Galunisertib Other: Laboratory Biomarker Analysis Drug: Paclitaxel	Phase 1	2016-03	Completed
NCT03911973	Phase 2 Trial With Safety Run-In of Gedatolisib Plus Talazoparib in Advanced Triple Negative or BRCA1/2 Positive, HER2 Negative Breast Cancers Big Ten Cancer Research Consortium BTCRC-BRE18-337	TNBC - Triple-Negative Breast Cancer	Drug: Gedatolisib Drug: Talazoparib	Phase 1 Phase 2	2019-04-17	Active, not recruiting
NCT01920061	A phase 1b open-label three-arm multicenter study to assess the safety and tolerability of pf-05212384 (pi3k/mtor inhibitor) in combination with other anti-tumor agents	Neoplasm	Drug: PF-05212384 (gedatolisib) Drug: Docetaxel Drug: Cisplatin Drug: Dacomitinib	Phase 1	2013-09-10	Completed
NCT03243331	An Initial Safety Study of Gedatolisib Plus PTK7-ADC for Metastatic Triple-negative Breast Cancer	Triple Negative Breast Cancer	Drug: Gedatolisib Drug: PTK7-ADC	Phase 1	2018-01-19	Completed

		Metastatic Breast Cancer				
NCT04266353	Mechanisms in IGF2 Induced Chemoresistance and Mitochondrial Regulation in Triple Negative Breast Cancer	Chemoprevention	Dietary Supplement: Resveratrol (RSV)	Not Applicable	2019-04-24	Withdrawn (Due to COVID-19)
NCT05846789	A Phase II Trial of Carboplatin +/- Tocilizumab As Initial Therapy for Metastatic Triple Negative and ER-low Breast Cancers	Metastatic Breast Cancer<br>Triple Negative Breast Cancer<br>Estrogen-receptor-low Breast Cancer	Drug: Carboplatin<br>Drug: Tocilizumab	Phase 2	2024-07-02	Recruiting

*These data were obtained from www.clinicaltrials.gov on May 27th, 2025, via the search term “TNBC” and the drugs selected in **Table S2**.
